# RmCP, a cerato-platanin protein from *Rigidoporus microporus*, induces defense responses during interaction with *Hevea brasiliensis*

**DOI:** 10.3389/fmicb.2025.1553350

**Published:** 2025-06-17

**Authors:** Nor Afiqah Maiden, Safiah Atan, Wong Mui-Yun

**Affiliations:** ^1^Production Development Division, Malaysian Rubber Board, Selangor, Malaysia; ^2^Institute of Plantation Studies, Universiti Putra Malaysia, Serdang, Selangor, Malaysia; ^3^Department of Plant Protection, Faculty of Agriculture, Universiti Putra Malaysia, Serdang, Selangor, Malaysia

**Keywords:** cerato-platanin, elicitor, plant immunity, *Rigidoporus microporus*, *Hevea brasiliensis*, white root disease

## Abstract

**Introduction:**

The rubber tree (*Hevea brasiliensis*) is susceptible to various fungal pathogens with *Rigidoporus microporus* being one of the most harmful. This fungus causes white root disease in rubber trees which can potentially lead to massive tree losses if left untreated. The use of elicitor proteins in enhancing host plant resistance represents a sustainable approach for disease control by reducing the use of chemical fungicides. Although cerato-platanin proteins (CPs) are recognized elicitors in many pathosystems, CP from *R. microporus* has not been functionally characterized, leaving its role in rubber–pathogen interactions unknown.

**Methods:**

The coding sequence of the CP homolog RmCP was heterologously expressed in *Escherichia coli* and purified to homogeneity by two-steps purification method, namely, affinity and size-exclusion chromatography. Bioactivity was assessed by infiltrating micromolar concentrations of RmCP into leaves of the host (*H. brasiliensis*) and a model non-host (*Nicotiana tabacum*).

**Results:**

Cell death (Trypan blue), reactive-oxygen species (DAB/NBT), callose deposition (aniline blue) and transcription of four defense-related genes (*HbCDPK5, HbMAPK, HbPR3, HbEDS1*) were monitored over 72 h. Purified RmCP migrated as a single band between 11 and 17 kDa band. Infiltration induced localized necrosis in *N. tabacum* within 48 h and in detached rubber leaves within 72 h. Both hosts accumulated H₂O₂ and O₂^−^, and deposited callose. Additionally, significant up-regulation of *HbCDPK5* and *HbMAPK* (early signaling), followed by strong induction of downstream effector genes, *HbPR3* and *HbEDS1* was observed in *H. brasiliensis*. These findings identify RmCP as the first basidiomycete CP shown to activate multilayer innate immunity in a latex-producing perennial.

**Conclusion:**

The study extends the functional spectrum of the CP family beyond ascomycete models and provides a biochemically defined platform for developing protein-based priming agents to combat white-root disease in rubber plantations.

## Introduction

White root disease, caused by the basidiomycete *Rigidoporus microporus*, is one of the most pervasive diseases of rubber. The rubber tree, or *Hevea brasiliensis*, is the only commercial producer of natural rubber worldwide, making it a plant of significant economic importance despite the fact that there are over 2,500 other species that can produce rubber (Yamashita and Takahashi, 2020; Amerik et al., 2021). Rubber white root disease may lead to reductions in land productivity and latex yield if it is not treated. Current control measures relies on the use of chemical fungicides which are environmentally damaging and uneconomical. Hence, sustainable plant defense strategies are urgently needed (Hamid and Wong, 2017; De Britto and Jogaiah, 2022; Li X. et al., 2022; Guo and Cheng, 2022; Luti et al., 2020; Baccelli, 2015; Gaderer et al., 2014; Pazzagli et al., 2014; Wang et al., 2018; Yang et al., 2018; Zhang et al., 2020; Zaparoli et al., 2009; Barsottini et al., 2013; Baccelli et al., 2015; Chen et al., 2015; Hamid et al., 2024; Maiden et al., 2024).

The ability of elicitors to activate or induce plant resistance makes them a key tool in the fight against plant diseases (Hamid and Wong, 2017) by priming plant’s innate immune system, thereby reducing disease severity and reliance on chemical fungicides (Frías et al., 2013; Yang et al., 2018; Quarantin et al., 2019; Rojas Moreno et al., 2023). Many investigations over the past 10 years have discovered elicitors produced from microbes that trigger immunological responses in many plant species (De Britto and Jogaiah, 2022; Li X. et al., 2022). The cerato-platanin family of proteins (CPP) is one of the most widely reported groups of elicitor proteins. CPPs are small proteins with four cysteine residues, protein lengths between 105 and 241 amino acids and a signature sequence of either CSD or CSN (Chen et al., 2013). The four cysteine residues form two intramolecular disulfide bridges which confers the stability of CPPs to high temperature and acidic media (De Oliveira et al., 2011; Pazzagli et al., 2014). Studies on the tertiary structure of CPPs from *Ceratocystis platani*, *Moniliophthora perniciosa* and *Trichoderma virens* revealed the presence of a double ψβ-barrel fold (De Oliveira et al., 2011; Barsottini et al., 2013).

Generally, CPPs are able to trigger plant’s immune response, hence, considered as microbe-associated molecular patterns (MAMPs; Gaderer et al., 2014; Pazzagli et al., 2014). Besides expression during interaction with plant host, expressions during hyphal growth, mycelial development, sporulation, and spore maturation have also been reported suggesting roles in fungal growth and development (Pan et al., 2018; Wang et al., 2018; Yang et al., 2018; Zhang et al., 2020). CPPs have also been reported to play role in fungal virulence (Zhang et al., 2017, 2020; Pan et al., 2018; Wang et al., 2018; Yang et al., 2018). Additionally, CPPs were reported to be able to protect fungal cell wall from enzymatic degradation (Quarantin et al., 2016; Zhang et al., 2017). Nevertheless, the most widely reported activity of CPPs was the ability to induce cell death and necrosis (Ashwin et al., 2017; Zhang et al., 2017, 2020; Li et al., 2019a, 2019b).

CPPs often exhibit capability to induce disease resistance against plant pathogen owing to their aptness in stimulating plant’s immune response. This function makes CPPs excellent candidates as priming agents especially since the protection conferred by CPPs appeared to be broad-spectrum (Yang et al., 2018; Yu et al., 2018; Li et al., 2019b; Quarantin et al., 2019). After more than 20 years since the discovery of the first member of the CPP family, the reports on functional studies of CPPs have mostly been focused on ascomycetes. Despite the presence of multiple copies of CPPs in basidiomycetes, the only functional studies to date were on CPPs from *Moniliophthora perniciosa*, *Heterobasidion annosum*, *Heterobasidion irregulare* and *Ganoderma boninense* (Zaparoli et al., 2009; Barsottini et al., 2013; Baccelli et al., 2015; Chen et al., 2015; Hamid et al., 2024).

A member of the cerato-platanin protein family, RmCP, from the rubber pathogen, *R. microporus* was previously isolated (Maiden et al., 2024). However, no functional study has been conducted to date on CPPs from *R. microporus*, and their biological roles in host-pathogen interaction remain unexplored. Consequently, this study characterizes the first cerato-platanin isolated from *R. microporus*, RmCP. RmCP was heterologously expressed in *Escherichia coli* and purified to homogeneity. Functional analysis revealed that RmCP triggered cell-death lesions, reactive-oxygen bursts, callose deposition, and induction of *HbCDPK5*, *HbMAPK*, *HbPR3* and *HbEDS1* in both host (*H. brasiliensis*) and non-host (*Nicotiana tabacum*) tissues. This study provides the first insight into CPP-mediated immunity in *Hevea*-*Rigidoporus* pathosystem and lays the groundwork for protein based priming strategies against white root disease.

## Materials and methods

### Fungal strain and plant material

The fungus *R. microporus* isolate AM, a virulent strain collected from Air Molek, Melaka, Malaysia, was obtained from the Integrated Pest and Disease Management Unit, Malaysian Rubber Board (MRB) and maintained on potato dextrose agar (PDA) at 25°C in the dark.

Seedlings of *H. brasiliensis* clone RRIM 2002 were purchased from Pendang Nursery Sdn. Bhd. (Pendang, Malaysia) and kept in a greenhouse under natural climatic conditions (25–37°C, 40–96% relative humidity) at the Sungai Buloh Research Station, MRB. Seeds of tobacco (*Nicotiana tabacum*) were kindly provided by Lembaga Kenaf dan Tembakau Negara (LKTN) and grown in the greenhouse.

### Heterologous expression of RmCP protein

The truncated CDS of RmCP, without signal peptide and stop codon, was cloned into the pEASY^®^-Blunt E2 vector (TransGen Biotech, China) and transformed into *E. coli Trans*B(DE3; TransGen Biotech, China) for expression of recombinant proteins. The expression of RmCP recombinant protein was induced with 0.3 mM of IPTG and shaking at 200 rpm for 18 h at 20°C. The expressed recombinant protein was purified from the soluble fraction using HisTrap™ HP affinity chromatography column (GE Healthcare Bio-Sciences AB, Sweden) coupled with ÄKTAprime plus liquid chromatography system (GE Healthcare, United States) and eluted through a gradient of 20 mM to 500 mM imidazole. Fractions corresponding to the desired peak were collected and further purified and desalted using HiPrep 16/60 Sephacryl S-200 High Resolution gel filtration chromatography column (GE Healthcare Bio-Sciences AB, Sweden). The purity and molecular weight (MW) of the recombinant RmCP protein were analyzed using SDS-PAGE.

### Cell-death inducing activity of RmCP

The effects of RmCP recombinant protein in host (*H. brasiliensis*) and non-host (*N. tabacum*) were assessed. Leaves of 4-weeks old *N. tabacum* were infiltrated with 20 μL purified RmCP protein on the abaxial side using sterile needle-less syringe. A volume of 20 μL of storage buffer (0.05 M sodium phosphate, 0.15 M NaCl, pH 7.2) was infiltrated as the negative control. The formation of necrotic lesion was observed daily. Different concentrations of purified RmCP (0, 1, 2, 3, and 4 μM) in a 20 μL volume were infiltrated into tobacco leaves to determine the minimum concentration of RmCP needed for necrosis.

Application of purified RmCP on *H. brasiliensis* was performed on detached leaves of *H. brasiliensis* clone RRIM 2002. Leaves at the limp green stage were collected and cleaned with sterile distilled water. The cleaned leaves were placed with the abaxial side facing up, in a Petri plate containing sterile filter paper soaked in sterile distilled water with a layer of parafilm on top of the filter paper. Subsequently, the leaf was pricked with sterile syringe needle and 20 μL of purified RmCP solution was dropped onto the area. For the negative control, 20 μL of storage buffer was used. The Petri plates were covered and incubated at room temperature (23–27°C). Formation of necrotic lesion was observed daily. *N. tabacum* leaves were collected at 24 h post infiltration (hpi) while leaves of *H. brasiliensis* were collected at 72 hpi for further analysis. All experiments were conducted with 5 replicates.

The induction of cell death was visualized microscopically through trypan blue staining. Trypan blue staining was conducted according to Fernández-Bautista et al. (2016) with slight modifications. Leaf tissues were immersed in 0.5% trypan blue stain solution (Nacalai Tesque, Japan) for an hour followed by rinsing in absolute ethanol. The leaf tissues were then placed in absolute ethanol and incubated overnight at room temperature with mild agitation. Thereafter, the ethanol solution was replaced with fresh absolute ethanol until all green tissue turned colorless. Completely cleared leaf tissues were viewed and photographed using Olympus CX43 biological microscope (Olympus Life Sciences, Japan).

### Accumulation of reactive oxygen species in leaves treated with RmCP

The generation of hydrogen peroxide (H_2_O_2_) and superoxide (O_2_^−^) in infiltrated tissues were assayed through histochemical staining using 3,3’-Diaminobenzidine (DAB) and nitro blue tetrazolium (NBT), respectively. DAB (Sigma-Aldrich, United States) and NBT (Sigma-Aldrich, United States) staining were performed according to Kumar et al. (2014) with slight modifications.

The infiltrated leaves were immersed in DAB solution (1 mg/mL, pH 3.8) or NBT solution (0.2% in 50 mM sodium phosphate buffer, pH 7.5) and incubated overnight in darkness at room temperature with mild agitation. The chlorophyll in the leaves were then cleared by boiling in absolute ethanol with several changes until the leaves became completely cleared. The accumulation of DAB and NBT deposits were then viewed and photographed with Olympus CX43 biological microscope (Olympus Life Sciences, Japan).

### Callose deposition in leaves treated with RmCP

Infiltrated leaves were stained with aniline blue (Sigma-Aldrich, United States) to visualize callose deposition. Staining was performed according to Schenk and Schikora (2015). Firstly, the leaf tissues were fixed and cleared overnight in acetic acid: ethanol (1:3) solution at room temperature with mild agitation. The leaves were then washed in 150 mM potassium phosphate dibasic (K_2_HPO_4_) for 30 min followed by incubation in aniline blue solution (0.01% in 150 mM K_2_HPO_4_) at room temperature in darkness for at least 2 h with mild agitation. The leaves were then directly viewed and photographed under UV filter with Olympus CX43 biological microscope (Olympus Life Sciences, Japan).

### Application of purified RmCP recombinant protein on detached *Hevea brasiliensis* leaves

Leaves of RRIM 2002 clones at the limp green stage were collected and cleaned with sterile distilled water and dried with sterile tissue paper. The leaves were then placed with the abaxial side facing upwards on top of a layer of parafilm on sterile filter paper moistened with sterile distilled water in Petri plates. The leaves were then pricked with sterile syringe needle at three different areas each along the right and left side on the leaf blade. A volume of 20 μL of purified recombinant RmCP protein was dropped onto each pricked area while 20 μL of storage buffer was used as the negative control. The Petri plates were covered and incubated at room temperature. The leaves were collected at 24, 48, and 72 h post application (hpa). Three biological replicates were prepared for each treatment at each sampling point. The mid veins were removed, and 500 mg of leaf tissue was weighed, wrapped in aluminum foil, and immediately frozen with liquid nitrogen. Samples were stored at −80°C.

### Total RNA extraction and cDNA synthesis

The collected and frozen leaf tissue was ground into powder using liquid nitrogen with mortar and pestle. The leaf powder was then added into a mixture of 3 mL extraction buffer (50 mM Tris–HCl, 150 mM LiCl, 5 mM EDTA, 5% SDS, pH 9.0), 3 mL chloroform, 120 μL *β*-mercaptoethanol and 120 mg polyvinylpyrrolidone (PVP), subsequently vortexed and incubated at room temperature for 5 min. The mixture was centrifuged for 15 min at 12,000 x *g* and 4°C. The top clear supernatant layer was transferred into a new microcentrifuge tube. Total RNA was extracted from the supernatant using TRIzol Reagent (Invitrogen, United States) according to manufacturer’s instructions. The obtained RNA pellet was resuspended in 50 μL RNase-free water. RNA clean-up was performed using RNeasy Plant Mini Kit (Qiagen, Germany) according to manufacturer’s instructions. Removal of genomic DNA was performed using RQ1 RNase-Free DNase (Promega, United States). The DNase I-treated total RNA samples were utilized in cDNA synthesis using RevertAid First Strand cDNA synthesis Kit (Thermo Scientific, United States).

### Reverse transcription quantitative real-time PCR analysis

Quantification of the expression level of four *H. brasiliensis* defense-related genes by RT-qPCR was performed using 1:10 dilution of cDNA as templates. Primers used for RT-qPCR are listed in Table 1. Amplification was conducted using CFX96™ Real-Time System (Bio-Rad, United States) and 1X *TransStart*® Tip Green qPCR SuperMix (TransGen Biotech, China). Reference genes, ubiquitin-protein ligases 2a (*HbUBC2a*) and ubiquitin-protein ligase 4 (*HbUBC4*) were employed as the internal controls. The relative fold-change expression level between treated and control sample was determined according to method described by Vandesompele et al. (2002) using CFX Maestro 2.2 software (Bio-Rad, United States). Statistical significance was analyzed using data from three biological replicates at a significant level (*α*) equal to 0.05 using a two-tailed Student’s t-test in CFX Maestro 2.2 (Bio-Rad, United States).

**Table 1:** List of primers used in RT-qPCR of *Hevea brasiliensis*.

Primer name	Primer sequence (5′–3′)	Target Gene	Source
*HbUBC2a*-F	CATTTATGCGGATGGAAGCA	ubiquitin-protein ligase 2a, *HbUBC2a*	Li et al. (2011)
*HbUBC2a*-R	CAGGGGAGTTTGGATTTGGA		
*HbUBC4*-F	TCCTTATGAGGGCGGAGTC	ubiquitin-protein ligase 4, *HbUBC4*	
*HbUBC4*-R	CAAGAACCGCACTTGAGGAG		
*HbCDPK5*-F	ATGGAGTTGCTCCTGATAGA	calcium-dependent protein kinase 5, *HbCDPK5*	XM_021804721.1
*HbCDPK5*-R	TGGAACATCTCTCGCAAAC		
*HbMAPK*-F	TACCGTGCACCAGAACTA	mitogen-activated protein kinase, *HbMAPK*	XM_021798013.1
*HbMAPK*-R	CTGATGGAGTCCCAATTAGC		
*HbPR3*-F	CAGCAGCCAAAGCATTTC	chitinase, *HbPR3*	XM_021790654.1
*HbPR3*-R	CTCCTCCTTGTGGCAATATC		
*HbEDS1*-F	GCTGTTCCCTAGTCTCAAAC	enhanced disease susceptibility 1, *HbEDS1*	XM_021815604.1
*HbEDS1*-R	CAAGAGAGTGCCCTGTAAAC		

## Results

### Heterologous expression and purification of RmCP

**Figure 1:**
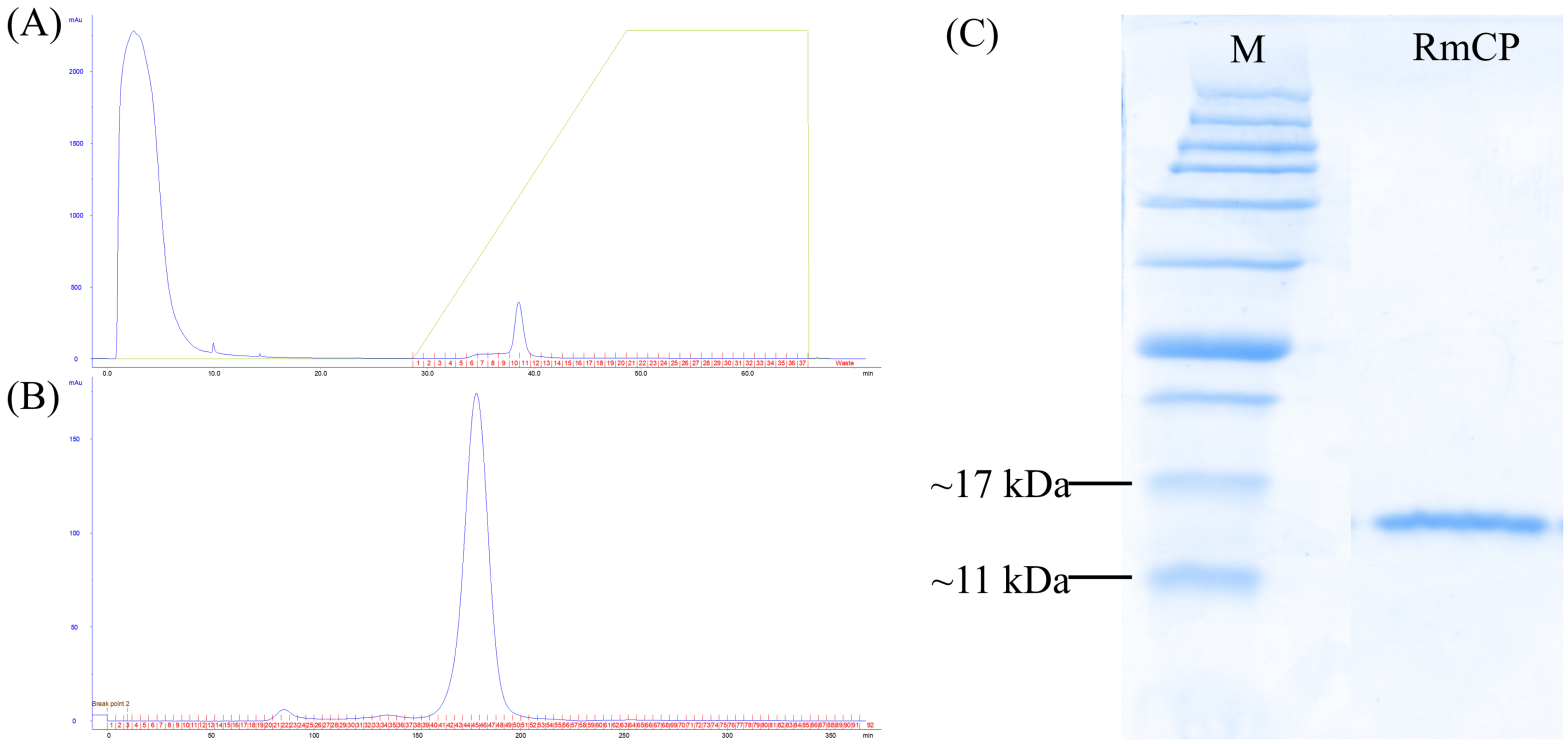
Purification of RmCP recombinant protein. **(A)** Elution profile of affinity chromatography using HisTrap™ HP affinity column (GE Healthcare Bio-Sciences AB, Sweden) coupled with ÄKTAprime plus system (GE Healthcare, United States) for the purification of RmCP from the soluble fraction of cell lysate. **(B)** Elution profile of gel filtration chromatography using HiPrep 16/60 Sephacryl S-200 High Resolution column (GE Healthcare Bio-Sciences AB, Sweden) coupled with ÄKTAprime plus system (GE Healthcare, USA) for the purification of RmCP from the pooled fractions from affinity chromatography. **(C)** SDS-PAGE analysis of purified RmCP. Clear migration of a single band of the His-tagged recombinant RmCP was observed between 11 kDa and 17 kDa.

The full-length coding sequence (CDS) of RmCP was amplified from *R. microporus* using RT-PCR. A fragment sized 438 bp encoding a protein with 145 a.a. residues was obtained. Purification of the histidine tagged RmCP recombinant protein using HisTrap™ HP affinity column (GE Healthcare Bio-Sciences AB, Sweden) coupled with ÄKTAprime plus system (GE Healthcare, United States) resulted in a single peak during gradient elution step with imidazole (Figure 1A). Further purification was conducted using HiPrep 16/60 Sephacryl S-200 High Resolution column (GE Healthcare Bio-Sciences AB, Sweden) for simultaneous purification and desalting of the sample. One high and sharp peak representing RmCP recombinant protein was observed with one small peak representing the co-eluted proteins (Figure 1B). The fractions were collected and pooled. The SDS-PAGE analysis of the pooled fraction showed migration of a clear single band between 17 kDa and 11 kDa indicating that the RmCP recombinant protein has been purified to homogeneity (Figure 1C).

### RmCP induces cell death, accumulation of reactive oxygen species, and callose deposition in *Nicotiana tabacum* and *Hevea brasiliensis*

**Figure 2:**
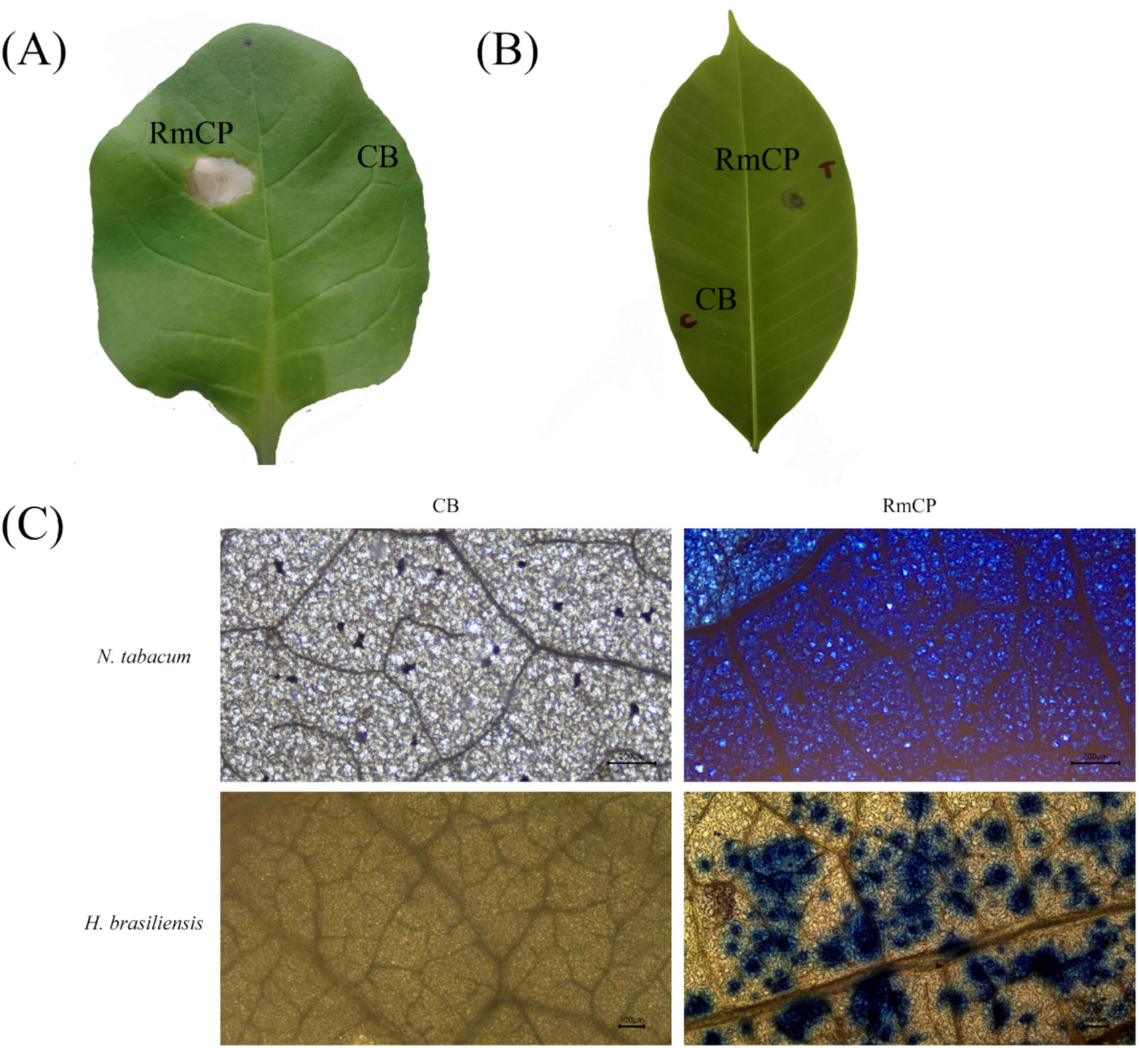
The cell death-inducing activity of RmCP. **(A)** *Nicotiana tabacum* leaf and **(B)** *Hevea brasiliensis* leaf were infiltrated with RmCP and control buffer (0.05 M sodium phosphate, 0.15 M NaCl, pH 7.2; CB) and photographed on the fifth day post infiltration (dpi). Leaf area infiltrated with RmCP displayed clear necrotic lesion while the area infiltrated with CB appeared clear of any lesion. **(C)** Microscopic observation of cell death in *N. tabacum* and *H. brasiliensis* leaves infiltrated with CB and RmCP. Leaves were stained with trypan blue at 24 h post infiltration (hpi) and observed under light microscope. Dead leaf cells were stained blue.

Many CPPs have been shown to have the ability to induce cell death in plants. *N. tabacum* is a well-established system for elicitor study, hence, the effects of RmCP recombinant protein were assayed on both the model (*N. tabacum*) and host (*H. brasiliensis*) plants. The effects of the purified protein were studied by applying the purified RmCP on the leaves. Infiltration of RmCP into leaves of *N. tabacum* resulted in cell death manifested as the formation of necrotic lesion observed on the second day post infiltration (dpi) which continued to enlarge up to the 5 dpi. No necrosis was observed at the site infiltrated with the control buffer (Figure 2A).

The application of RmCP on detached *H. brasiliensis* leaves also resulted in the formation of necrotic lesion whereas the control buffer did not have any visible effects (Figure 2B). The formation of necrosis was observed on the third day post application (dpa) and continued to develop up to the seventh dpa.

The infiltrated leaf samples were stained with Trypan blue to observe cell death microscopically. The infiltrated areas in *N. tabacum* and *H. brasileinsis* leaves were stained blue indicating cell death. On the contrary, the area infiltrated with control buffer was not stained (Figure 2C).

**Figure 3:**
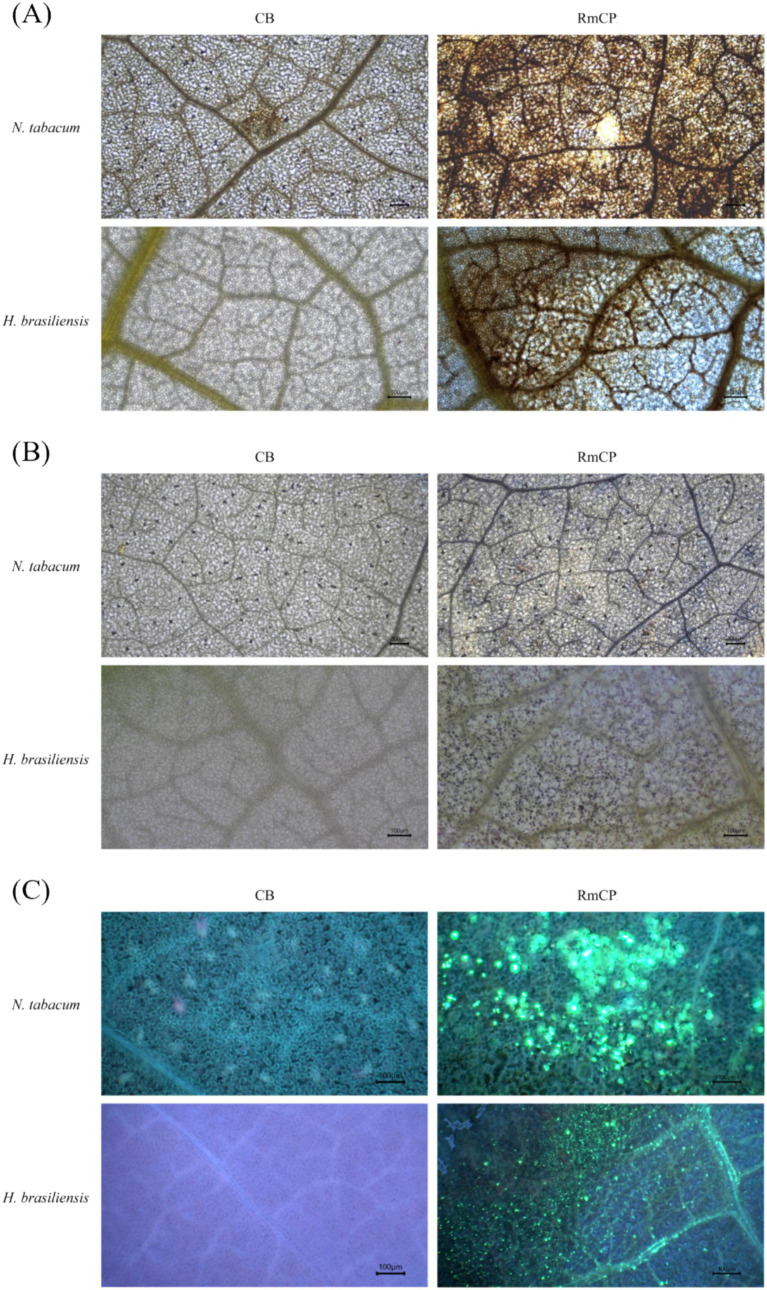
The accumulation of reactive oxygen species (ROS) and callose deposition in infiltrated *N. tabacum* and *H. brasiliensis* leaves. **(A)** Microscopic observation of H_2_O_2_ accumulation in *N. tabacum* and *H. brasiliensis* leaves. Leaves infiltrated with control buffer (0.05 M sodium phosphate, 0.15 M NaCl, pH 7.2; CB) and RmCP were stained with 3,3′-diaminobenzidine (DAB) 24 h post infiltration (hpi) and photographed under light microscope. H_2_O_2_ accumulation indicated by brown DAB deposits observed in leaves infiltrated with RmCP but not in CB. **(B)** Microscopic observation of O_2_^−^ accumulation in *N. tabacum* and *H. brasiliensis* leaves. Infiltrated leaves were stained with nitro blue tetrazolium (NBT). O_2_^−^ accumulation indicated by blue precipitates of formazan observed in leaves infiltrated with RmCP but not in CB. **(C)** Microscopic observation of callose deposition in *N. tabacum* and *H. brasiliensis* leaves. Infiltrated leaves were stained with aniline blue and photographed under light microscope with UV filter. Callose deposition indicated by green fluorescence observed in leaves infiltrated with RmCP but not in CB.

The generation of ROS, specifically H_2_O_2_ and O_2_^−^, was assayed in plant cells infiltrated with RmCP. Staining using DAB on the infiltrated leaves of both tobacco and rubber exhibited brown precipitates which signified the presence of H_2_O_2_ (Figure 3A). The staining of the leaves with NBT exhibited blue deposits indicating the formation of formazan resulting from the reaction between NBT and O_2_^−^ (Figure 3B). The staining of tobacco and rubber leaves with aniline blue exhibited fluorescent green deposits indicating the presence of callose (Figure 3C). On the contrary, infiltration with control buffer did not produce any fluorescent green deposits.

### RmCP activates the expression of defense-related genes in *Hevea brasiliensis*

**Figure 4:**
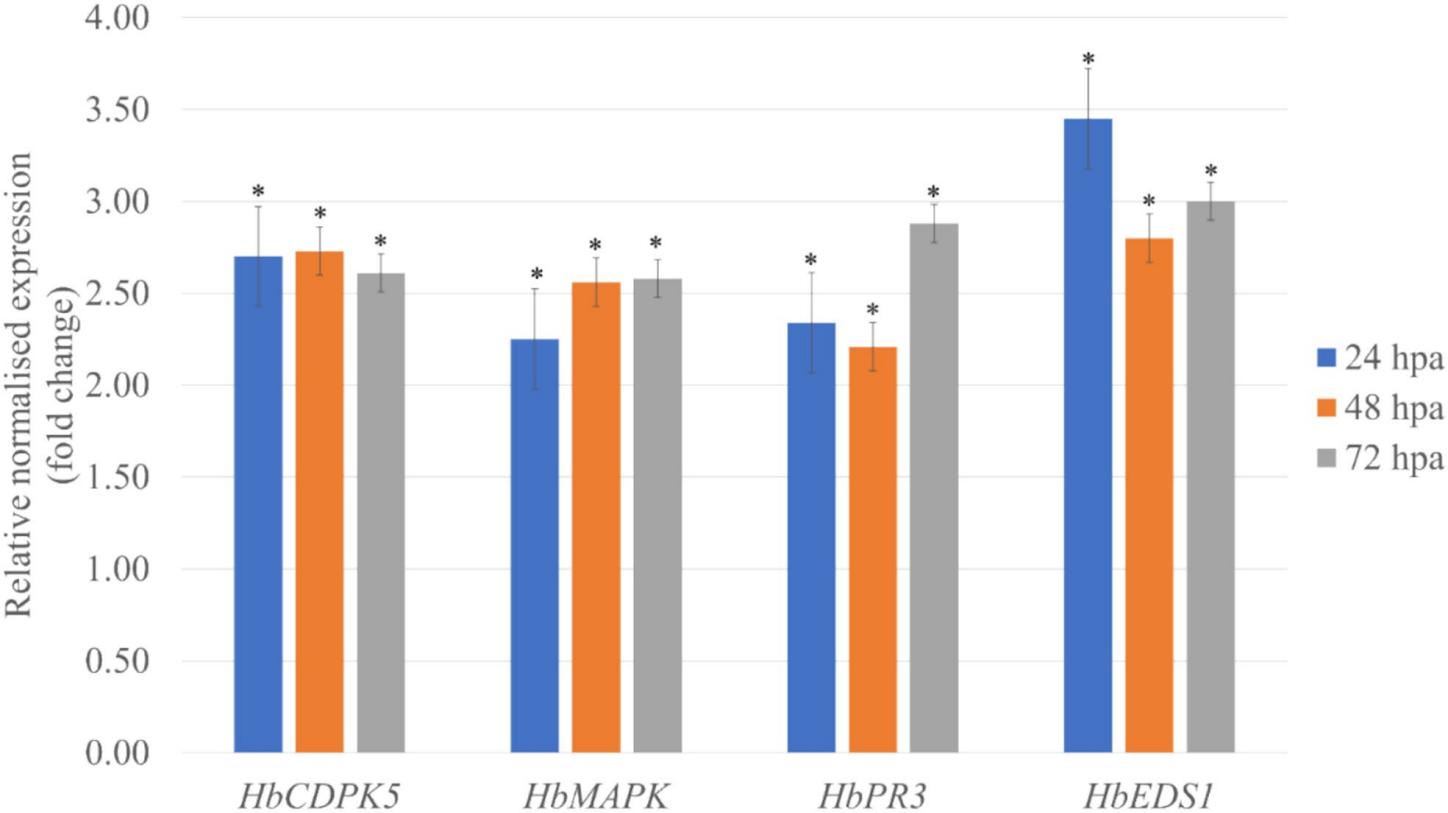
Relative abundance of *HbCDPK5* (calcium-dependent protein kinase 5), *HbMAPK* (mitogen-activated protein kinase), *HbPR3* (chitinase), and *HbEDS1* (enhanced disease susceptibility 1) transcripts in leaves treated with purified RmCP recombinant protein. *HbUBC2a* (ubiquitin-protein ligase 2a) and *HbUBC4* (ubiquitin-protein ligase 4) were used as internal control to normalize the data. The relative transcriptional abundances of the defense-related genes were determined after 24, 48 and 72 h post application (hpa) of RmCP and compared to expression level in control leaves treated with control buffer (0.05 M sodium phosphate, 0.15 M NaCl, pH 7.2). Error bars were calculated based on three replicates. Asterisks indicate significant differences (*p < 0.05, t-test*).

Reverse Transcription Quantitative Real Time PCR (RT-qPCR) was utilized to determine the expression of four defense-related genes in leaves of *H. brasiliensis* treated with purified RmCP recombinant protein. These defense-related genes include calcium-dependent protein kinase 5 (*HbCDPK5*), mitogen-activated protein kinase (*HbMAPK*), chitinase (*HbPR3*), and enhanced disease susceptibility 1 (*HbEDS1*). The RT-qPCR results showed significantly elevated expression of all defense-genes in *H. brasiliensis* leaves treated with RmCP compared to control throughout all tested time points (Figure 4).

## Discussion

Cerato-platanin proteins have been studied mainly in ascomycete fungi, leaving the functional role of their basidiomycete counterparts largely unexplored (Oghenekaro et al., 2020; Shao et al., 2021; Prasad et al., 2022; Ghozlan et al., 2020; Liao et al., 2022; Li et al., 2020; Liao et al., 2022; McCombe et al., 2022; Ngou et al., 2022; Shamrai, 2022; Mansoor et al., 2022; Castro et al., 2021; Lukan and Coll, 2022; Singh et al., 2021; Pan et al., 2018; Wang et al., 2018; Li et al., 2019a; Quarantin et al., 2019; Zhang et al., 2020; Wang et al., 2021; Wang et al., 2013, 2016; Li et al., 2019a; Bredow and Monaghan, 2019; Dekomah et al., 2022; Zhang et al., 2022; Li L. et al., 2022; Sun and Zhang, 2022; Oghenekaro et al., 2016; Ribeiro et al., 2021; Ali et al., 2018; Boccardo et al., 2019; Chouhan et al., 2022; Kaur et al., 2022; Chiu et al., 2022; Vaghela et al., 2022; Oghenekaro et al., 2016; Woraathasin et al., 2017; Syafaah et al., 2020; Bernacki et al., 2019; Wang et al., 2020; Dongus and Parker, 2021; Ramírez-Zavaleta et al., 2022; Cheng et al., 2018; Li et al., 2019a). Our investigation identifies RmCP from the basidiomycete *R. microporus* as a potent elicitor that orchestrates multi-layered defense responses in its host, *H. brasiliensis,* and non-host, the model plant *N. tabacum*. The recombinant protein, obtained as a single 11–17 kDa band, produced rapid necrosis in *Nicotiana tabacum* within 48 h and in detached rubber leaves within 72 h, accompanied by a burst of hydrogen peroxide, superoxide and extensive callose deposition. These responses mirror but also extend the spectrum of activities reported for ascomycete CPPs such as FgCPP2, SsCP1, cmcp and FocCP1 (Yang et al., 2018; Li et al., 2019a; Quarantin et al., 2019; Zhang et al., 2020), which trigger hypersensitive-like cell death yet seldom have been evaluated in woody hosts. Our data therefore demonstrates that CPP-mediated defense is not restricted to annual model plants and highlights RmCP as the first basidiomycete CPP shown to activate multilayered innate immunity in a commercially important tree crop.

One of the most widely reported activity of CPPs was the ability to induce cell death and necrosis. The formation of necrotic lesions was observed after the application of RmCP on tobacco and rubber leaves indicating that the purified RmCP recombinant protein was functionally active and was able to trigger cell death. Cell death was also observed microscopically with Trypan blue staining. *R. microporus* is a necrotrophic fungus which kills the host’s living cells and feeds on dead tissues (Oghenekaro et al., 2020). While cell death is beneficial to impede the advancement of biotrophic or hemibiotrophic pathogens, the effects on necrotrophs is the opposite as necrotrophs co-opt host programmed cell death (PCD; Shao et al., 2021; Prasad et al., 2022). Infection by necrotrophic pathogens involves the secretion of phytotoxins, cell wall degrading enzymes (CWDEs), cell death-inducing proteins (CDIPs), and other extracellular enzymes resulting in necrotic lesions in the host (Ghozlan et al., 2020; Liao et al., 2022). CDIPs, also known as necrosis-inducing proteins (NIPs), are cytotoxic to dicotyledonous plants and secreted by a wide range of microbes. It has been demonstrated that CDIPs can cause cell death, promote pathogen colonization, and induce plant immune response, hence, considered as pathogen-associated molecular patterns (PAMPs; Li et al., 2020; Liao et al., 2022). Therefore, the ability of RmCP to induce cell death implies a possible role as CDIPs, secreted during infection process to promote host colonization.

The recognition of pathogen by plant receptors triggers an array of defense reactions including the accumulation of ROS, the production of antimicrobial compounds and defense-related hormones, callose deposition, the influx of Ca^2+^ into the cytosol, stomatal closure, transcriptional reprogramming, and hypersensitive response (HR) that results in localized plant cell death (McCombe et al., 2022; Ngou et al., 2022; Shamrai, 2022). The ability of RmCP to trigger plant immune responses including accumulation of ROS, callose deposition and expression of defense-related genes was evaluated.

The accumulation of ROS, specifically hydrogen peroxide (H_2_O_2_) and superoxide anion (O_2_^.-^), were observed in tobacco and rubber leaves following application of RmCP. These two forms of ROS are some of the most stable and abundant ROS in plants (Mansoor et al., 2022). The production of ROS is crucial for plant development and response to abiotic and biotic stress (Castro et al., 2021) and represents one of the earliest hallmarks of plant immune response (Lukan and Coll, 2022). The functions of ROS during host and pathogen interactions include roles in the mediation of cell wall modifications, the regulation of different plant hormone signaling pathways and the induction of PCD (Singh et al., 2021). The accumulation of ROS was reported in multiple studies on CPPs (Pan et al., 2018; Wang et al., 2018; Li et al., 2019a; Quarantin et al., 2019; Zhang et al., 2020).

Another component of defense response toward pathogen attack is callose deposition. Callose deposition was observed in tobacco and rubber leaves infiltrated with RmCP. The deposition of callose between the plasma membrane and the cell wall, at the plasmodesmata, and on other plant tissues is intended to slow the invasion and spread of pathogens (Wang et al., 2021). Several studies on CPPs have also reported callose deposition in infiltrated leaves (Wang et al., 2013, 2016; Li et al., 2019a). Given that callose plugs are pivotal for restricting the advance of pathogens in trees, the robust deposition we recorded implies that RmCP may enhance physical barriers against *R. microporus* spread in the field.

The expression of four defense-related genes, *HbCDPK5*, *HbMAPK*, *HbPR3*, and *HbEDS1* were assessed following application of purified RmCP recombinant protein on *H. brasiliensis* leaves. Transcript analysis corroborated the biochemical hallmarks of immunity. Up-regulated expressions of *HbCDPK5*, *HbMAPK*, *HbPR3*, and *HbEDS1* compared to control were observed throughout all sampling points. RmCP triggered the rapid Ca^2+^ influx that was indirectly detected through *HbCDPK5* induction. CDPKs act as sensor proteins with four EF-hand motifs and an autoinhibitory junction domain which enables CDPKs to translate transient cytosolic Ca^2+^ spikes into phosphorylation of NADPH oxidase (Rboh) and attendant ROS production (Bredow and Monaghan, 2019; Dekomah et al., 2022). Consistent with that role, *HbCDPK5* transcripts peaked at 24 h post-application, the same window in which DAB/NBT staining revealed H₂O₂ and O₂^−^ accumulation. *HbCDPK5* was reported to regulate the accumulation of ROS in rubber tree mesophyll protoplast and its’ overexpression in *Arabidopsis* enhanced the resistance to *Botrytis cinerea* indicating roles in plant disease resistance (Zhang et al., 2022).

Downstream of this Ca^2+^ node, the *HbMAPK* gene showed a sustained two- to three-fold up-regulation through 72 h. MAPKs contains the conserved TEY activation loop and were reported to play multiple roles in plant defense including the phosphorylation of WRKY transcription factors related to disease resistance, regulation of the synthesis of plant antitoxins, mediation of cell wall thickening through callose deposition, activation of plant hypersensitivity through overexpression of defense related genes and oxidative burst, promotion of stomatal closure to impede pathogen invasion and lastly involvement in the synthesis of plant disease resistance-related hormones (Li L. et al., 2022; Sun and Zhang, 2022). The temporal separation between CDPK5 and MAPK induction suggests a relay in which Ca^2+^ sensors ignite a MAPK cascade that maintains defense momentum after the initial ROS burst subsides. Similar to this study, the expression of MAPKs was reported to be up regulated in rubber shoot inoculated with *R. microporus* (Oghenekaro et al., 2016) and in rubber leaves treated with purified cassiicolin Cas1, an effector from the rubber leaf pathogen, *Corynespora cassiicola* (Ribeiro et al., 2021).

Beyond initiating signaling events, RmCP also drives the upregulation of key effector genes. Pathogenesis-related (PR) proteins is a group of functionally diverse proteins produced in response to a pathogen attack, with roles in limiting the growth and spread of pathogens (Ali et al., 2018). PR proteins can directly affect the pathogens, and/or generate signal molecules to induce other plant defense related pathways (Boccardo et al., 2019). PR proteins accumulate locally inside the infected as well as distant un-infected tissues, hence, preventing the spread of infection and leading to systemic acquired resistance (Chouhan et al., 2022; Kaur et al., 2022). The *PR3* gene encodes a class-III chitinase whose (*β*/*α*)₈ TIM-barrel fold and catalytic Glu-123/Glu-125 dyad acting as hydrolytic enzymes which are able to digest chitin, the structural element of fungal cell wall, therefore, effective against a wide range of phytopathogenic fungi (Chiu et al., 2022; Vaghela et al., 2022). Its robust expression coincides with visible callose deposition, implying a coordinated fortification of the cell wall. Several studies have reported the up regulation of *HbPR3* in rubber tissue inoculated with *R. microporus* as observed in this study (Oghenekaro et al., 2016; Woraathasin et al., 2017; Syafaah et al., 2020).

Enhanced Disease Susceptibility 1 (EDS1), a lipase-like α/β hydrolase that scaffolds PAD4-SAG101 complexes is a key component involved in resistance (R) gene-mediated and basal disease resistance specifically in signal transduction toward transcriptional reprogramming and cell death (Bernacki et al., 2019; Wang et al., 2020; Dongus and Parker, 2021; Ramírez-Zavaleta et al., 2022). The expression of *HbEDS1* remained elevated throughout the time course, consistent with its role in sustaining systemic acquired resistance beyond the local hypersensitive response. In accordance with the findings in this study, the application of CPPs Epl1 from *Trichoderma formosa* and FocCP1 from *Fusarium oxysporum* were also reported to result in the up regulation of *EDS1* in tobacco (Cheng et al., 2018; Li et al., 2019a).

Collectively, these structural and temporal patterns outline the following model: RmCP surface loops act as MAMPs, triggering Ca^2+^ influx. CDPK5 then phosphorylates Rboh to produce an oxidative burst that both restricts pathogen ingress and feeds forward into the MAPK module. The MAPK-WRKY pathway amplifies defense gene activation, notably *HbPR3*, while the EDS1 ensures long-term salicylic acid dependent immunity. This integrated response explains the convergence of ROS accumulation, callose deposition and cell-death observed in detached-leaf assays and underscores RmCP’s dual potential as both virulence factor and deployable elicitor for priming rubber trees against white-root disease.

The findings of this study position RmCP as the first experimentally validated cerato-platanin from a necrotrophic basidiomycete pathogen of rubber. By bridging the longstanding knowledge gap between the abundance of CP homologs in basidiomycetes and their largely unexplored biological roles, our results not only extend the functional spectrum of the CP protein family beyond ascomycete models but also establishes a solid foundation, with biochemical and defense data, for future research and practical applications. Results from this study point to a signaling network capable of amplifying local alerts into systemic readiness. Harnessing RmCP or engineered peptide derivatives that retain defense inducing activity but lack necrotic side-effects, could provide an eco-friendly and broad-spectrum priming agent to bolster rubber-tree resilience against white-root disease and other fungal threats.

## Data Availability

The datasets presented in this study can be found in online repositories. The names of the repository/repositories and accession number(s) can be found at: https://www.ncbi.nlm.nih.gov/genbank/, OR880706.

## Glossary

CDIP: Cell death-inducing protein
CDS: Coding sequence
CPP: Cerato-platanin protein
CWDE: Cell wall-degrading enzymes
DAB: 3,3’-Diaminobenzidine
dpi: Days post infiltration
dpa: Days post application
hpa: Hours post application
hpi: Hours post infiltration
HR: Hypersensitive response
MAMP: Microbe-associated molecular pattern
MRB: Malaysian Rubber Board
MW: Molecular weight
NBT: Nitro blue tetrazolium
NIP: Necrosis-inducing protein
PAMP: Pathogen-associated molecular pattern
PCD: Programmed cell death
PDA: Potato dextrose agar
PVP: Polyvinylpyrrolidone
ROS: Reactive oxygen species
**RT-qPCR**: Reverse transcription quantitative real time PCR

## References

[ref1] Ali S. Ganai B. A. Kamili A. N. Bhat A. A. Mir Z. A. Bhat J. A.. (2018). Pathogenesis-related proteins and peptides as promising tools for engineering plants with multiple stress tolerance. Microbiol. Res. 212-213, 29–37. doi: 10.1016/J.MICRES.2018.04.008, PMID: 29853166

[ref2] Amerik A. Y. Martirosyan Y. T. Martirosyan L. Y. Goldberg V. M. Uteulin K. R. Varfolomeev S. D. (2021). Molecular genetic analysis of natural rubber biosynthesis. Russ. J. Plant Physiol. 68, 31–45. doi: 10.1134/S1021443721010039

[ref3] Ashwin N. M. R. Barnabas L. Ramesh Sundar A. Malathi P. Viswanathan R. Masi A.. (2017). Comparative secretome analysis of Colletotrichum falcatum identifies a cerato-platanin protein (EPL1) as a potential pathogen-associated molecular pattern (PAMP) inducing systemic resistance in sugarcane. J. Proteome 169, 2–20. doi: 10.1016/j.jprot.2017.05.020, PMID: 28546091

[ref4] Baccelli I. (2015). Cerato-platanin family proteins: one function for multiple biological roles? Front. Plant Sci. 5, 2013–2016. doi: 10.3389/fpls.2014.00769, PMID: 25610450 PMC4284994

[ref5] Baccelli I. Gonthier P. Bernardi R. (2015). Gene expression analyses reveal a relationship between conidiation and cerato-platanin in homokaryotic and heterokaryotic strains of the fungal plant pathogen Heterobasidion irregulare. Mycol. Prog. 14, 1–8. doi: 10.1007/s11557-015-1063-x

[ref6] Barsottini M. R. De Oliveira J. F. Adamoski D. Teixeira P. J. P. L. Do Prado P. F. V. Tiezzi H. O.. (2013). Functional diversification of cerato-platanins in moniliophthora perniciosa as seen by differential expression and protein function specialization. Mol. Plant-Microbe Interact. 26, 1281–1293. doi: 10.1094/MPMI-05-13-0148-R 23902259

[ref7] Bernacki M. J. Czarnocka W. Szechyńska-Hebda M. Mittler R. Karpiński S. (2019). Biotechnological potential of LSD1, EDS1, and PAD4 in the improvement of crops and industrial plants. Plan. Theory 8:290. doi: 10.3390/plants8080290, PMID: 31426325 PMC6724177

[ref8] Boccardo N. A. Segretin M. E. Hernandez I. Mirkin F. G. Chacón O. Lopez Y.. (2019). Expression of pathogenesis-related proteins in transplastomic tobacco plants confers resistance to filamentous pathogens under field trials. Sci. Rep. 9, 1–13. doi: 10.1038/s41598-019-39568-6, PMID: 30808937 PMC6391382

[ref9] Bredow M. Monaghan J. (2019). Regulation of plant immune signaling by calcium-dependent protein kinases. Mol. Plant-Microbe Interact. 32, 6–19. doi: 10.1094/MPMI-09-18-0267-FI, PMID: 30299213

[ref10] Castro B. Citterico M. Kimura S. Stevens D. M. Wrzaczek M. Coaker G. (2021). Stress-induced reactive oxygen species compartmentalization, perception and signalling. Nat Plants 7, 403–412. doi: 10.1038/s41477-021-00887-0, PMID: 33846592 PMC8751180

[ref11] Chen H. Kovalchuk A. Keriö S. Asiegbu F. O. (2013). Distribution and bioinformatic analysis of the cerato-platanin protein family in Dikarya. Mycologia 105, 1479–1488. doi: 10.3852/13-115, PMID: 23928425

[ref12] Chen H. Quintana J. Kovalchuk A. Ubhayasekera W. Asiegbu F. O. (2015). A cerato-platanin-like protein HaCPL2 from Heterobasidion annosum sensu stricto induces cell death in Nicotiana tabacum and *Pinus sylvestris*. Fungal Genet. Biol. 84, 41–51. doi: 10.1016/j.fgb.2015.09.007, PMID: 26385823

[ref13] Cheng C. H. Shen B. N. Shang Q. W. Liu L. Y. D. Peng K. C. Chen Y. H.. (2018). Gene-to-gene network analysis of the mediation of plant innate immunity by the eliciting plant response-like 1 (Epl1) elicitor of trichoderma formosa. Mol. Plant-Microbe Interact. 31, 683–691. doi: 10.1094/MPMI-01-18-0002-TA, PMID: 29436965

[ref14] Chiu T. Poucet T. Li Y. (2022). The potential of plant proteins as antifungal agents for agricultural applications. Synth Syst Biotechnol 7, 1075–1083. doi: 10.1016/J.SYNBIO.2022.06.009, PMID: 35891944 PMC9305310

[ref15] Chouhan R. Ahmed S. Gandhi S. G. (2022). Over-expression of PR proteins with chitinase activity in transgenic plants for alleviation of fungal pathogenesis. J. Plant Pathol., 105:1–13. doi: 10.1007/S42161-022-01226-8/TABLES/1

[ref16] De Britto S. Jogaiah S. (2022). Priming with fungal elicitor elicits early signaling defense against leaf spot of broccoli underlying cellular, biochemical and gene expression. Microbiol. Res. 263:127143. doi: 10.1016/J.MICRES.2022.127143, PMID: 35944354

[ref17] De Oliveira A. L. Gallo M. Pazzagli L. Benedetti C. E. Cappugi G. Scala A.. (2011). The structure of the elicitor cerato-platanin (CP), the first member of the CP fungal protein family, reveals a double ψβ-barrel fold and carbohydrate binding. J. Biol. Chem. 286, 17560–17568. doi: 10.1074/jbc.M111.223644, PMID: 21454637 PMC3093830

[ref18] Dekomah S. D. Bi Z. Dormatey R. Wang Y. Haider F. U. Sun C.. (2022). The role of CDPKs in plant development, nutrient and stress signaling. Front. Genet. 13:2799. doi: 10.3389/fgene.2022.996203 PMC9561101 36246614

[ref19] Dongus J. A. Parker J. E. (2021). EDS1 signalling: at the nexus of intracellular and surface receptor immunity. Curr. Opin. Plant Biol. 62:102039. doi: 10.1016/J.PBI.2021.102039, PMID: 33930849

[ref20] Fernández-Bautista N. Domínguez-Núñez J. Moreno M. M. Berrocal-Lobo M. (2016). Plant tissue trypan blue staining during phytopathogen infection. Bioanalysis 6, 1–7. doi: 10.21769/bioprotoc.2078

[ref21] Frías M. Brito N. González C. (2013). The Botrytis cinerea cerato-Platanin BcSpl1 is a potent inducer of systemic acquired resistance (SAR) in tobacco and generates a wave of salicylic acid expanding from the site of application. Mol. Plant Pathol. 14, 191–196. doi: 10.1111/j.1364-3703.2012.00842.x, PMID: 23072280 PMC6638659

[ref22] Gaderer R. Bonazza K. Seidl-Seiboth V. (2014). Cerato-platanins: a fungal protein family with intriguing properties and application potential. Appl. Microbiol. Biotechnol. 98, 4795–4803. doi: 10.1007/s00253-014-5690-y, PMID: 24687753 PMC4024134

[ref23] Ghozlan M. H. EL-Argawy E. Tokgöz S. Lakshman D. K. Mitra A. Ghozlan M. H. (2020). Plant defense against necrotrophic pathogens. Am. J. Plant Sci. 11, 2122–2138. doi: 10.4236/AJPS.2020.1112149

[ref24] Guo J. Cheng Y. (2022). Advances in fungal elicitor-triggered plant immunity. Int. J. Mol. Sci. 23:12003. doi: 10.3390/ijms231912003, PMID: 36233304 PMC9569958

[ref25] Hamid S. Ho C. L. Abdullah S. N. A. Low E. T. L. Nagappan J. Wong M. Y. (2024). Characterisation and expression analyses of two putative cerato-platanin proteins isolated from *Ganoderma boninense*. Pat. Physiol. Mol. Plant Pathol. 131, 1–12. doi: 10.1016/j.pmpp.2024.102289

[ref26] Hamid S. Wong M.-Y. (2017). Elicitors and their roles in plant Defence against pathogens particularly Basidiomycetes. Crop. Improv., 305–334. doi: 10.1007/978-3-319-65079-1_14

[ref27] Kaur S. Samota M. K. Choudhary M. Choudhary M. Pandey A. K. Sharma A.. (2022). How do plants defend themselves against pathogens-biochemical mechanisms and genetic interventions. Physiol. Mol. Biol. Plants 28, 485–504. doi: 10.1007/s12298-022-01146-y, PMID: 35400890 PMC8943088

[ref28] Kumar D. Yusuf M. A. Singh P. Sardar M. Sarin N. B. (2014). Histochemical detection of superoxide and H2O2 accumulation in *Brassica juncea* seedlings. Bioanalysis 4, 1–4. doi: 10.21769/BioProtoc.1108

[ref29] Li S. Dong Y. Li L. Zhang Y. Yang X. Zeng H.. (2019a). The novel cerato-platanin-like protein FocCP1 from Fusarium oxysporum triggers an immune response in plants. Int. J. Mol. Sci. 20, 1–19. doi: 10.3390/ijms20112849, PMID: 31212693 PMC6600160

[ref30] Li Y. Han Y. Qu M. Chen J. Chen X. Geng X.. (2020). Apoplastic cell death-inducing proteins of filamentous plant pathogens: roles in plant-pathogen interactions. Front. Genet. 11, 1–20. doi: 10.3389/fgene.2020.00661, PMID: 32676100 PMC7333776

[ref31] Li X. Liu M. Liu Y. Zhao W. Li S. Liu W.. (2022). A putative effector of the rubber-tree powdery mildew fungus has elicitor activity that can trigger plant immunity. Planta 255, 33–13. doi: 10.1007/s00425-021-03818-7, PMID: 34997357

[ref32] Li S. Nie H. Qiu D. Shi M. Yuan Q. (2019b). A novel protein elicitor PeFOC1 from Fusarium oxysporum triggers defense response and systemic resistance in tobacco. Biochem. Biophys. Res. Commun. 514, 1074–1080. doi: 10.1016/j.bbrc.2019.05.018, PMID: 31097222

[ref33] Li H. Qin Y. Xiao X. Tang C. (2011). Screening of valid reference genes for real-time RT-PCR data normalization in Hevea brasiliensis and expression validation of a sucrose transporter gene HbSUT3. Plant Sci. 181, 132–139. doi: 10.1016/J.PLANTSCI.2011.04.014, PMID: 21683878

[ref34] Li L. Zhu X.-M. Zhang Y.-R. Cai Y.-Y. Wang J.-Y. Liu M.-Y.. (2022). Research on the molecular interaction mechanism between plants and pathogenic Fungi. Int. J. Mol. Sci. 23:4658. doi: 10.3390/IJMS23094658 35563048 PMC9104627

[ref35] Liao C. J. Hailemariam S. Sharon A. Mengiste T. (2022). Pathogenic strategies and immune mechanisms to necrotrophs: differences and similarities to biotrophs and hemibiotrophs. Curr. Opin. Plant Biol. 69:102291. doi: 10.1016/J.PBI.2022.102291 36063637

[ref36] Lukan T. Coll A. (2022). Intertwined roles of reactive oxygen species and salicylic acid signaling are crucial for the plant response to biotic stress. Int. J. Mol. Sci. 23:5568. doi: 10.3390/ijms23105568, PMID: 35628379 PMC9147500

[ref37] Luti S. Sella L. Quarantin A. Pazzagli L. Baccelli I. (2020). Twenty years of research on cerato-platanin family proteins: clues, conclusions, and unsolved issues. Fungal Biol. Rev. 34, 13–24. doi: 10.1016/j.fbr.2019.10.001

[ref38] Maiden N. A. Atan S. Syd Ali N. Ahmad K. Wong M.-Y. (2024). The cerato-platanin gene, rmcp, from *Rigidoporus microporus* was stably expressed during infection of *Hevea brasiliensis*. J. Rubber Res. 27, 1–10. doi: 10.1007/s42464-024-00253-7

[ref39] Mansoor S. Wani O. A. Lone J. K. Manhas S. Kour N. Alam P.. (2022). Reactive oxygen species in plants: from source to sink. Antioxidants 11:225. doi: 10.3390/antiox11020225, PMID: 35204108 PMC8868209

[ref40] McCombe C. L. Greenwood J. R. Solomon P. S. Williams S. J. (2022). Molecular plant immunity against biotrophic, hemibiotrophic, and necrotrophic fungi. Essays Biochem. 66, 581–593. doi: 10.1042/EBC20210073, PMID: 35587147 PMC9528087

[ref41] Ngou B. P. M. Ding P. Jones J. D. G. (2022). Thirty years of resistance: zig-Zag through the plant immune system. Plant Cell 34, 1447–1478. doi: 10.1093/plcell/koac041, PMID: 35167697 PMC9048904

[ref42] Oghenekaro A. O. Kovalchuk A. Raffaello T. Camarero S. Gressler M. Henrissat B.. (2020). Genome sequencing of Rigidoporus microporus provides insights on genes important for wood decay, latex tolerance and interspecific fungal interactions. Sci. Rep. 10, 5250–5215. doi: 10.1038/s41598-020-62150-4, PMID: 32251355 PMC7089950

[ref43] Oghenekaro A. O. Omorusi V. I. Asiegbu F. O. (2016). Defence-related gene expression of *Hevea brasiliensis* clones in response to the white rot pathogen, *Rigidoporus microporus*. For. Pathol. 46, 318–326. doi: 10.1111/efp.12260

[ref44] Pan Y. Wei J. Yao C. Reng H. Gao Z. (2018). SsSm1, a Cerato-platanin family protein, is involved in the hyphal development and pathogenic process of Sclerotinia sclerotiorum. Plant Sci. 270, 37–46. doi: 10.1016/j.plantsci.2018.02.001, PMID: 29576085

[ref45] Pazzagli L. Seidl-Seiboth V. Barsottini M. Vargas W. A. Scala A. Mukherjee P. K. (2014). Cerato-platanins: elicitors and effectors. Plant Sci. 228, 79–87. doi: 10.1016/j.plantsci.2014.02.009, PMID: 25438788

[ref46] Prasad L. Katoch S. Shahid S. (2022). Microbial interaction mediated programmed cell death in plants. Biotech 12, 1–18. doi: 10.1007/s13205-021-03099-7 35096500 PMC8761208

[ref47] Quarantin A. Castiglioni C. Schäfer W. Favaron F. Sella L. (2019). The Fusarium graminearum cerato-platanins loosen cellulose substrates enhancing fungal cellulase activity as expansin-like proteins. Plant Physiol. Biochem. 139, 229–238. doi: 10.1016/j.plaphy.2019.03.025, PMID: 30913532

[ref48] Quarantin A. Glasenapp A. Schäfer W. Favaron F. Sella L. (2016). Involvement of the Fusarium graminearum cerato-platanin proteins in fungal growth and plant infection. Plant Physiol. Biochem. 109, 220–229. doi: 10.1016/j.plaphy.2016.10.001, PMID: 27744264

[ref49] Ramírez-Zavaleta C. Y. García-Barrera L. J. Rodríguez-Verástegui L. L. Arrieta-Flores D. Gregorio-Jorge J. (2022). An overview of PRR- and NLR-mediated immunities: conserved signaling components across the plant kingdom that communicate both pathways. Int. J. Mol. Sci. 23:12974. doi: 10.3390/IJMS232112974/S1, PMID: 36361764 PMC9654257

[ref50] Ribeiro S. Label P. Garcia D. Montoro P. Pujade-Renaud V. (2021). Transcriptome profiling in susceptible and tolerant rubber tree clones in response to cassiicolin Cas1, a necrotrophic effector from Corynespora cassiicola. PLoS One 16:e0254541. doi: 10.1371/JOURNAL.PONE.0254541, PMID: 34320014 PMC8318233

[ref51] Rojas Moreno M. M. González-Pérez E. Rodríguez-Hernandez A. A. Ortega-Amaro M. A. Becerra-Flora A. Serrano M.. (2023). Expression of EPL1 from Trichoderma atroviride in Arabidopsis confers resistance to bacterial and fungal pathogens. Plants (Basel) 12, 1–16. doi: 10.3390/plants12132443, PMID: 37447005 PMC10347261

[ref52] Schenk S. T. Schikora A. (2015). Staining of callose deposition in root and leaf tissues. Bio Protoc. 5, 1–4. doi: 10.21769/BioProtoc.1429

[ref53] Shamrai S. M. (2022). Recognition of pathogen attacks by plant immune sensors and induction of plant immune response. Cytol. Genet. 56, 46–58. doi: 10.3103/S0095452722010108

[ref54] Shao D. Smith D. L. Kabbage M. Roth M. G. (2021). Effectors of plant necrotrophic Fungi. Front. Plant Sci. 12:995. doi: 10.3389/FPLS.2021.687713/BIBTEX PMC8213389 34149788

[ref55] Singh Y. Nair A. M. Verma P. K. (2021). Surviving the odds: from perception to survival of fungal phytopathogens under host-generated oxidative burst. Plant Commun 2:100142. doi: 10.1016/J.XPLC.2021.100142, PMID: 34027389 PMC8132124

[ref56] Sun T. Zhang Y. (2022). MAP kinase cascades in plant development and immune signaling. EMBO Rep. 23:e53817. doi: 10.15252/EMBR.202153817, PMID: 35041234 PMC8811656

[ref57] Syafaah A. Woraathakorn N. Plodpai P. Nualsri C. Nakkanong K. (2020). Comparative growth performance and activity of defense related enzymes and gene expression in rubber clones against *Rigidoporus microporus* infection. Pak. J. Biotechnol. 17, 161–172. doi: 10.34016/PJBT.2020.17.3.161

[ref58] Vaghela B. Vashi R. Rajput K. Joshi R. (2022). Plant chitinases and their role in plant defense: a comprehensive review. Enzym. Microb. Technol. 159:110055. doi: 10.1016/J.ENZMICTEC.2022.110055, PMID: 35537378

[ref59] Vandesompele J. De Preter K. Pattyn F. Poppe B. Van Roy N. De Paepe A.. (2002). Accurate normalization of real-time quantitative RT-PCR data by geometric averaging of multiple internal control genes. Genome Biol. 3:RESEARCH0034. doi: 10.1186/GB-2002-3-7-RESEARCH0034, PMID: 12184808 PMC126239

[ref60] Wang W. An B. Feng L. He C. Luo H. (2018). A Colletotrichum gloeosporioides cerato-platanin protein, CgCP1, contributes to conidiation and plays roles in the interaction with rubber tree. Can. J. Microbiol. 64, 826–834. doi: 10.1139/cjm-2018-0087, PMID: 29870670

[ref61] Wang W. Feng B. Zhou J. Tang D. (2020). Plant immune signaling: advancing on two frontiers. J. Integr. Plant Biol. 62, 2–24. doi: 10.1111/jipb.12898, PMID: 31846204

[ref62] Wang Y. Li X. Fan B. Zhu C. Chen Z. (2021). Regulation and function of defense-related callose deposition in plants. Int. J. Mol. Sci. 22, 1–15. doi: 10.3390/ijms22052393 PMC7957820 33673633

[ref63] Wang Y. Song J. Wu Y. Odeph M. Liu Z. Howlett B. J.. (2013). Eplt4 Proteinaceous elicitor produced in Pichia pastoris has a protective effect against Cercosporidium sofinum infections of soybean leaves. Appl. Biochem. Biotechnol. 169, 722–737. doi: 10.1007/s12010-012-0015-z, PMID: 23271623

[ref64] Wang Y. Wu J. Kim S. G. Tsuda K. Gupta R. Park S. Y.. (2016). Magnaporthe oryzae-secreted protein MSP1 induces cell death and elicits defense responses in rice. Mol. Plant-Microbe Interact. 29, 299–312. doi: 10.1094/MPMI-12-15-0266-R, PMID: 26780420

[ref65] Woraathasin N. Nakkanong K. Nualsri C. (2017). Cloning and expression analysis of HbPR-1b and HbPR-3 in *hevea brasiliensis* during inoculation with *Rigidoporus microporus*. Pak. J. Biol. Sci. 20, 233–243. doi: 10.3923/pjbs.2017.233.243, PMID: 29023035

[ref66] Yamashita S. Takahashi S. (2020). Molecular mechanisms of natural rubber biosynthesis. Annu. Rev. Biochem. 89, 821–851. doi: 10.1146/annurev-biochem-013118-111107, PMID: 32228045

[ref67] Yang G. Tang L. Gong Y. Xie J. Fu Y. Jiang D.. (2018). A cerato-platanin protein SsCP1 targets plant PR1 and contributes to virulence of Sclerotinia sclerotiorum. New Phytol. 217, 739–755. doi: 10.1111/nph.14842, PMID: 29076546

[ref68] Yu W. Mijiti G. Huang Y. Fan H. Wang Y. Liu Z. (2018). Functional analysis of eliciting plant response protein Epl1-Tas from Trichoderma asperellum ACCC30536. Sci. Rep. 8, 7974–7913. doi: 10.1038/s41598-018-26328-1, PMID: 29789617 PMC5964103

[ref69] Zaparoli G. Cabrera O. G. Medrano F. J. Tiburcio R. Lacerda G. Pereira G. G. (2009). Identification of a second family of genes in Moniliophthora perniciosa, the causal agent of witches’ broom disease in cacao, encoding necrosis-inducing proteins similar to cerato-platanins. Mycol. Res. 113, 61–72. doi: 10.1016/j.mycres.2008.08.004, PMID: 18796332

[ref70] Zhang Y. Gao Y. Liang Y. Dong Y. Yang X. Yuan J.. (2017). The verticillium dahliae snodprot1-like protein VdCP1 contributes to virulence and triggers the plant immune system. Front. Plant Sci. 8, 1–13. doi: 10.3389/fpls.2017.01880, PMID: 29163605 PMC5671667

[ref71] Zhang Z. Li Y. Luo L. Hao J. Li J. (2020). Characterization of cmcp gene as a pathogenicity factor of *Ceratocystis manginecans*. Front. Microbiol. 11, 1–11. doi: 10.3389/fmicb.2020.01824 32849428 PMC7411389

[ref72] Zhang B. Song Y. Zhang X. Wang Q. Li X. He C.. (2022). Identification and expression assay of calcium-dependent protein kinase family genes in Hevea brasiliensis and determination of HbCDPK5 functions in disease resistance. Tree Physiol. 42, 1070–1083. doi: 10.1093/treephys/tpab156, PMID: 35022787

